# Supplementary material for: Direct Cost Analysis of Microbial Keratitis in North China: A Hospital-Based Retrospective Study
Source: Pathogens. 2024 Aug 7;13(8):666. doi: 10.3390/pathogens13080666 (PMC11357569; doi:10.3390/pathogens13080666)
Supplement: Supplementary file 1 [file pathogens-13-00666-s001.zip › pathogens-3115745-supplementary.pdf]

Table S1. Different types of mixed microbial keratitis (MMK)

Type of MMK	Number of cases
BK+FK	108
BK+VK	9
BK+AK	15
FK+AK	1

Note: BK, Bacterial Keratitis; FK, Fungal Keratitis; VK, Viral Keratitis; AK, Acanthamoeba Keratitis; MMK, Mixed Microbial Keratitis.

Table S2. Microorganism culture test results found in the samples

Pathogens	Number of cases
**Bacterial**	**79**
**Gram-positive**	**48**
Staphylococcus epidermidis	13
Bacillus megaterium	7
Streptococcus oralis	7
Streptococcus spp.	3
Staphylococcus aureus	3
Micrococcus luteus	2
Staphylococcus aureus subsp. *aureus*	2
Bacillus spp.	2
Staphylococcus capitis	1
Corynebacterium diphtheriae	1
Staphylococcus haemolyticus	1
Nocardia spp.	1
Bacillus cereus	1
Streptococcus agalactiae	1
Staphylococcus saprophyticus	1
Enterococcus faecalis	1
Streptococcus sanguinis	1
**Gram-negative**	**31**
Pseudomonas aeruginosa	6
Moraxella osloensis	5
Pseudomonas putida	3
Acinetobacter baumannii	3
Lactobacillus spp.	2
Enterobacter cloacae subsp. *cloacae*	2
Klebsiella oxytoca	2
Streptococcus intermedius	1
Nocardia otitidiscaviarum	1
Acinetobacter junii	1

Bacteroides fragilis	1
Neisseria gonorrhoeae	1
Moraxella nonliquefaciens	1
Escherichia coli	1
Proteus spp.	1
**Fungal**	**113**
**Mold**	**105**
Fusarium spp.	37
Fusarium solani	15
Ramularia spp.	10
Alternaria spp.	5
Candida parapsilosis	4
Alternaria alternata	4
Chaetomium spp.	4
Aspergillus spp.	3
Aspergillus flavus	3
Pleospora spp.	2
Penicillium spp.	2
Aspergillus versicolor	2
Aspergillus fumigatus	2
Alternaria tenuissima	2
Candida albicans	5
Candida spp.	1
Candida tropicalis	1
Aspergillus terreus	1
Aspergillus sydowii	1
Aspergillus lentulus	1
**Yeast**	**8**
Saccharomyces spp.	1
Penicillium citrinum	1
Fusarium verticilloides	1
Fusarium proliferatum	1
Fusarium oxysporum	1
Fusarium graminearum	1
Trichophyton spp.	1
Beauveria spp.	1
**Viral**	**11**
Herpes Simplex Virus Type I	5
Epstein-Barr virus	4
Varicella-Zoster virus	1
Adenovirus	1
**Parasite**	**48**

Acanthamoeba spp.	48
**Total**	**251**

Note: Calculated based on the number of patients with positive culture results or positive PCR results for viruses.

Table S3. The median outpatient and inpatient costs for patients with different types of keratitis.

**Outpatient costs**					
**Costs Type**	**BK**	**FK**	**VK**	**AK**	**MMK**
**Registration**	92.4 (56.0-161.7)	79.8 (50.4-126.0)	100.8 (67.2-168.0)	117.6 (72.1-254.8)	102.2 (54.6-159.6)
**Ophthalmic Exams**	49.3 (30.5-79.4)	53.8 (30.1-82.6)	62.6 (47.4-92.1)	127.8 (58.6-194.4)	73.5 (39.8-105.8)
**Lab Tests**	105.7 (95.9-199.6)	150.5 (95.9-231.0)	174.0 (95.9-211.4)	230.2 (128.8-348.3)	186.6 (105.7-268.9)
**Medications**	113.8 (53.8-258.4)	217.3 (131.4-379.3)	229.8 (127.6-400.8)	181.6 (60.6-398.3)	206.0 (63.5-450.9)
**Inpatient costs**					
**Costs Type**	**BK**	**FK**	**VK**	**AK**	**MMK**
**Registration**	84.0 (66.5-125.3)	98.0 (14.0-112.0)	91.0 (77.0-140.0)	63.0 (56.0-96.6)	78.4 (14.0-98.0)
**Ophthalmic Exams**	67.3 (35.5-135.6)	58.6 (0.0-107.3)	63.3 (51.9-136.1)	110.0 (60.6-126.4)	49.7 (20.9-87.1)
**Lab Tests**	76.6 (13.3-125.5)	21.5 (2.2-90.7)	161.7 (86.8-276.9)	80.0 (3.3-138.9)	81.2 (2.8-103.5)
**Medication**	274.7 (110.5-400.7)	520.7 (266.2-1298.4)	213.3 (212.1-256.5)	274.7 (202.6-340.4)	353.1 (235.1-573.2)
**Cornea Surgery**	505.1 (355.9-807.3)	675.2 (35.8-801.1)	689.2 (681.1-846.3)	758.6 (706.7-1036.9)	650.3 (313.2-829.7)
**Medical Consumables**	3230.4 (233.8-3316.2)	3276.4 (37.3-3324.3)	3289.8 (3203.6-3347.9)	3283.8 (3256.1-3318.7)	3282.8 (1582.2-3353.5)
**Minor Procedures**	30.6 (26.4-41.2)	33.5 (7.9-39.7)	46.2 (32.9-48.3)	24.4 (20.4-32.6)	26.2 (10.6-33.2)
**Other costs**	50.1 (35.7-77.0)	50.1 (7.1-60.4)	46.6 (41.9-77.6)	37.7 (29.8-50.0)	41.9 (8.3-58.4)

Note: BK, Bacterial Keratitis; FK, Fungal Keratitis; VK, Viral Keratitis; AK, Acanthamoeba Keratitis; MMK, Mixed Microbial Keratitis. Values are presented in US$ as median (IQR)
